# Increase of plasma IL-9 and decrease of plasma IL-5, IL-7, and IFN-γ in patients with chronic heart failure

**DOI:** 10.1186/1479-5876-9-28

**Published:** 2011-03-21

**Authors:** Claudia Cappuzzello, Luca Di Vito, Roberta Melchionna, Guido Melillo, Lorena Silvestri, Eleonora Cesareo, Filippo Crea, Giovanna Liuzzo, Antonio Facchiano, Maurizio C Capogrossi, Monica Napolitano

**Affiliations:** 1 Laboratorio di Biologia Vascolare e Medicina Rigenerativa, Centro Cardiologico Monzino-IRCCS, Milan, Italy; 2 Institute of Cardiology, Catholic University, Rome, Italy; 3 Laboratorio di Patologia Vascolare, Istituto Dermopatico dell'Immacolata-IRCCS, Rome, Italy; 4 Laboratorio di Analisi, Istituto Dermopatico dell'Immacolata-IRCCS, Rome, Italy; 5 Laboratorio di Ingegneria tissutale e Fisiopatologia cutanea, Istituto Dermopatico dell'Immacolata-IRCCS, Rome, Italy

## Abstract

**Background:**

Several cytokines are associated with the development and/or progression of chronic heart failure (CHF). Our aim was to look more closely at the cytokine networks involved in CHF, and to assess whether disease etiology affects cytokine expression. The study population was comprised of a) 69 patients with stable CHF, New York Heart Association (NYHA) II/IV classes, secondary to ischaemic (ICM) and non ischaemic dilated (NIDCM) cardiomyopathy and b) 16 control subjects. We analyzed and compared the plasma levels of 27 pro- and anti-inflammatory mediators, in the study population and assessed for any possible correlation with echocardiographic parameters and disease duration.

**Methods:**

27 cytokines and growth factors were analyzed in the plasma of ICM- (n = 42) and NIDCM (n = 27) NYHA class II-IV patients vs age- and gender-matched controls (n = 16) by a beadbased multiplex immunoassay. Statistical analysis was performed by ANOVA followed by Tukey post-hoc test for multiple comparison.

**Results:**

Macrophage inflammatory protein (MIP)-1β, Vascular endothelial growth factor (VEGF), interleukin (IL)-9, Monocyte chemotactic protein (MCP)-1, and IL-8 plasma levels were increased in both ICM and NIDCM groups vs controls. In contrast, IL-7, IL-5, and Interferon (IFN)-γ were decreased in both ICM and NIDCM groups as compared to controls. Plasma IL-6 and IL-1 β were increased in ICM and decreased in NIDCM, vs controls, respectively.

IL-9 levels inversely correlated, in ICM patients, with left ventricular ejection fraction (LVEF) while IL-5 plasma levels inversely correlated with disease duration, in NYHA III/IV ICM patients.

This is the first time that both an increase of plasma IL-9, and a decrease of plasma IL-5, IL-7 and IFN-γ have been reported in ICM as well as in NIDCM groups, vs controls. Interestingly, such cytokines are part of a network of genes whose expression levels change during chronic heart failure. The altered expression levels of MIP-1 β, VEGF, MCP-1, IL-1 β, IL-6, and IL-8, found in this study, are in keeping with previous reports.

**Conclusions:**

The increase of plasma IL-9, and the decrease of plasma IL-5, IL-7 and IFN-γ in ICM as well as in NIDCM groups vs controls may contribute to get further insights into the inflammatory pathways involved in CHF.

## Background

The role of inflammation in the pathogenesis and progression of chronic heart failure (CHF) is well established [1,2]. Typical hallmarks for the involvement of immune mechanisms in CHF pathogenesis are the activation/migration of leukocytes from the circulation to the areas of myocardial inflammation and an increased expression of proinflammatory cytokines, such as tumor necrosis factor α, interleukin-1, and interleukin-6 from a damaged myocardium [3-6]. In heart failure such cytokine levels increase in association with disease severity and may contribute to impair cardiac function through cardiomyocyte apoptosis, inflammatory response, cardiac hypertrophy and matrix metalloproteinase activation [1,7-9].

Proinflammatory cytokines may cause myocyte apoptosis and necrosis; interleukin-6 induces a hypertrophic response in myocytes [10], whereas TNF-α causes left ventricular dilatation, apparently through the activation of matrix metalloproteinases [11]. Elevated levels of CXC and C-C chemokines such as GROα, IL-8, MCP-1, RANTES and MIP-1α were also found in the most severe cases of heart failure, which would suggest a possible role for such molecules in CHF progression [12,13]. Peripheral blood mononuclear cells are important players in the CHF inflammatory process [14,15]. However, the mechanisms that, in heart disease, ultimately cause the transition from cardiac disease to heart failure still need further investigation.

The identification of the cytokines associated with chronic heart failure is not only important to get deeper insights into the inflammatory pathways involved, but it could also lead to the identification of disease's biomarkers. The biomarkers found in heart failure include BNP, NT pro-BNP, C-reactive protein, tumor necrosis factor α, interleukins 1 and 6, TNF-α, matrix metalloproteinases, neuroepinephrine, renin, galectin-3 [16-18], some of which are also important tools in the diagnosis and pathogenesis of heart failure, in the identification of subjects at risk of heart failure, risk stratification, therapeutic agents response monitoring [17].

The aim of this study was to investigate the inflammatory pathways associated with CHF, and to assess whether disease etiology affects cytokine cascades. To this end, we analyzed plasma cytokine levels in patients with stable ischaemic or non ischaemic dilated cardiomyopathy CHF vs healthy donors, by a multiplex cytokine/growth factor immunoassay, and then assessed their relationship to echocardiographic parameters and disease duration.

## Materials and methods

### Subjects

Sixty-nine patients with chronic stable heart failure for six consecutive months were enrolled at the Cardiology Unit of the Catholic University of Sacred Heart of Rome out of 210 patients admitted to the unit during the same period. Baseline patient demographic variables, heart failure classification and echocardiographic parameters of patients are shown in Table 1. CHF patients were classified in New York Heart Association (NYHA) functional class II/IV. The causes of heart failure were classified as 1) ICM in patients (n = 42) with a history of myocardial infarction and coronary atherosclerosis with a stenosis >70% in at least one major coronary artery branch or 2) NIDCM in patients (n = 27) with no history of myocardial infarction and angiografically normal coronary arteries. NIDCM is defined as patients with dilated cardiomyopathy, with LVEF less than 40%, in the absence of ischaemic or congenital heart disease, or cardiac valve disease. LVEF and left ventricular internal diastolic diameter (LVIDd) were calculated from 2-D and M-mode transthoracic echocardiographic images. Duration of disease was calculated as the time between the first ICM or NIDCM diagnosis and enrolment in our study. Exclusion criteria were infections, malignancies, autoimmune disorders, diabetes, pulmonary disease, myocardial infarction, unstable angina or myocarditis during the 6 months prior to enrollment in the study and blood sample collection. Sixteen age- and sex-matched controls were recruited at the Clinical Laboratory at IDI-IRCCS from randomly chosen individuals with no overt cardiac disease and dyslipidemia. Inflammatory indexes, i.e. C-reactive protein (CRP) and erytrocyte sedimentation rate (ESR), were within physiologic ranges and no leukocytosis was observed.

**Table 1:** Patient characteristics

	ICM (n = 42)	NIDCM (n = 27)
Age mean (range)	67 ± 7.4 (47-81)	65 ± 8.7 (47-80)
Sex M/F	41/1	24/3
NYHA class	II-IV	II-IV
LVEF %	33 ± 8.0	28 ± 7.0
LVIDd mm	62 ± 8.83	69 ± 9.53
**Medications %**		
Β-Antagonists	86	92
ACE-inhibitors	58	75
Loop diuretics	70	13
Aldosterone antagonists	34	46
Digitoxin	4	21*
Nitrate	9	3

* p < 0.05 vs ICM

The study complies with The Helsinki Declaration and was approved by the Ethics Committee of the Catholic University and all subjects gave written informed consent.

### Sample collection and management

Venous blood samples (20 ml) from ICM and NIDCM patients and controls were collected in pyrogen-free, heparinized tubes between 8.00 am and 10.00 am and immediately centrifuged at 3000 rpm for 15 minutes. Plasma samples were aliquoted and stored at -80°C. Samples were frozen and thawed only once.

### Multiplex immunoassay

We used a human cytokine 27-Bio-Plex assay kit (BioRad Laboratories, Milan, Italy), a beadbased multiplex immunoassay. This technology has the capacity to measure several cytokines/cytokine receptors and growth factors simultaneously in small volumes of plasma or serum with a greater detection dynamic range (~ 1-10,000 pg/mL) than single ELISAs [15-18] with high accuracy and sensitivity [19-22].

The factors analysed were: IL1-β, IL-1 receptor antagonist (ra), IL-2, IL-4, IL-5, IL-6, IL-7, IL-8, IL-9, IL-10, IL-12, IL-15, IL-16, 1L-17, basic fibroblast growth factor (FGF), eotaxin, granulocyte colony stimulating factor (G-CSF), granulocyte-macrophage colony stimulating factor (GM-CSF), IFN-γ, Interferon γ-inducible protein (IP)-10, MCP-1, MIP-1α, MIP-1 β, Platelet-derived growth factor (PDGF)-BB, Regulated on Activation Normal T-cell-Expressed and Secreted (RANTES), Tumor necrosis factor (TNF) α and VEGF. The lower detection limit was 0.2-19.3 pg/ml (see http://www.bio-rad.com/webroot/web/pdf/lsr/literature/Bulletin_3157.pdf). We tested the plasma concentrations of the above molecules in the whole study population. The plasma samples were diluted 1:4 and tested in duplicates. The samples were read on a Bio-Plex 200 instrument equipped with the software Bioplex Manager, version 4.1, using a five-parameters not-linear regression formula to compute sample concentrations from the standard curves.

The pubgene program http://www.pubgene.org was used to evaluate the expression and/or functional correlation between cytokines and growth factors.

### Statistical analysis

All the major study variables evaluated in the present study were not normally distributed; thus logarithmic transformation was applied to the data to allow parametric techniques to be used. The Bivariate Pearson correlation coefficient (r) was calculated between cytokine plasma levels and echocardiografic parameters or disease duration. Statistical analysis between ICM, NIDCM and CTRL was performed by ANOVA followed by Tukey's post-hoc test for multiple comparison. No significant differences were detected in the three groups in terms of age and gender (chi-square p value >0.05). The cytokines and growth factor values have been expressed as median and interquartile range and a p value <0.05 was considered statistically significant. Proportions (Table 1), were compared by Fisher's exact test.

## Results

### Plasma cytokine profiles in ICM and NIDCM CHF patients vs controls

Multiplex immunoassay screening for cytokine and growth factor protein expression, by means of the multiplex XMAP technology, was performed on ICM and NIDCM CHF patients (Table 1) and on control plasma.

**Figure 1:**
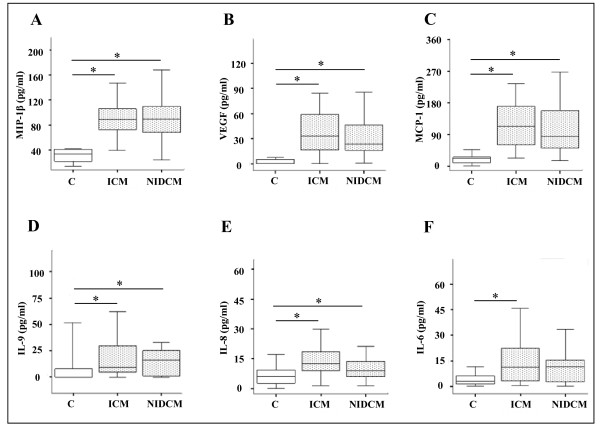
**Cytokine plasma levels increased in ICM and NIDCM CHF patients vs controls**. Plasma levels of MIP-1 β (A), VEGF (B), MCP-1 (C), IL-9 (D), IL-8 (E), and IL-6 (F) in patients with chronic heart failure (ICM n = 42; NIDCM n = 27) classified in NYHA class II-IV and in 16 healthy controls. Data are shown as "box-and-whiskers" plots (medians, 25^th ^to 75^th ^percentiles. * p < 0.05 vs controls.

The first group of factors was composed of MIP-1 β, VEGF, MCP-1, IL-9, and IL-8, (Figure 1A-E); the levels of these cytokines were increased in patients with ICM as well as in NIDCM with respect to controls (p < 0.05). Further, the IL-6 cytokine (panel F) was significantly increased in ICM patients vs healthy controls (p < 0.05). The cytokines and growth factor values have been expressed as median and interquartile range.

**Figure 2:**
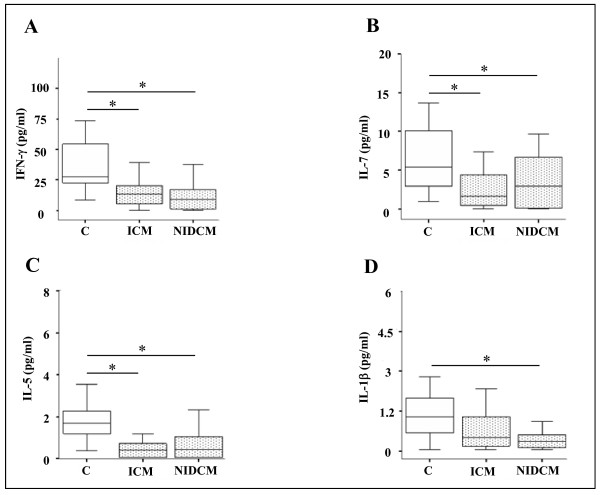
**Cytokine plasma levels decreased in ICM and NIDCM CHF patients vs controls**. Plasma levels of IFN-γ (A), IL-7 (B), IL-5 (C) and IL-1 β (D) in patients with chronic heart failure (ICM n = 42; NIDCM n = 27) classified in NYHA class II/IV and in 16 healthy controls. Data are given as medians and 25^th ^to 75^th ^percentile. * p < 0.05 vs controls.

The second group included IFN-γ, IL-7, and IL-5, whose levels were significantly lower in the plasma of ICM and NIDCM patients vs healthy individuals, see Figure 2 (panel A-C). IL-1 β, on the other hand, was only decreased in the NIDCM patients vs controls (Figure 2 panel D) (*p < 0.05).

In our study, several factors, i.e. MIP-1 β, VEGF, MCP-1, IL-8 and IL-6 were found to be modulated, in CHF patients, in accordance to previous reports [9,19]. Only a few of the 27 cytokines and growth factors, i.e. IL-2, IL-15, IL-17, bFGF, and MIP-1α showed undetectable levels in all three groups, except for sporadic cases. All other detectable molecules, except the ones describedFigure 1 and 2 showed no statistically significant variation between either ICM or NIDCM groups, and controls (data not shown).

However, what was noteworthy, and described here for the first time, was the increase of IL-9, and decrease of IL-5, IL-7 and IFN-γ plasma levels in patients with chronic heart failure.

**Figure 3:**
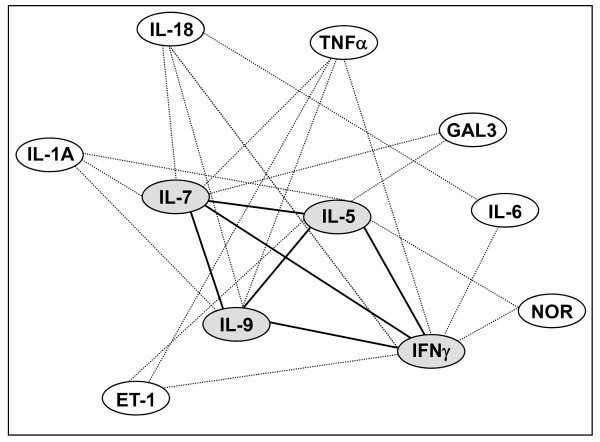
**Gene networks in CHF**. The lines link genes whose expression or function is affected by neighbouring genes. The gene network has been generated using the PUBGENE program http://www.pubgene.org. White ovals: genes previously known to be modulated in CHF. Grey ovals: genes found to be regulated in CHF in the present study. Endothelin-1 (ET-1), Galectin 3 (GAL3), Norepinephrine (NOR), Tumor necrosis factor (TNF) α, Interferon (IFN)- γ, Interleukin (IL) 1A, 5, 6, 7, 9, 18.

Using the bioinformatic analysis from the http://www.pubgene.org program, we showed, in Figure 3 that the molecules newly associated with chronic heart failure (gray ovals), are linked to each other in terms of expression and/or function (black thick lines), and to a number of molecules, namely Norepinephrine, Endothelin-1, IL-1A, Galectin-3, TNFα, IL-6 and IL-18 [13], which play a key role in CHF (white ovals).

### Correlation of cytokine plasma leves with echocardiographic parameters and disease duration

In order to assess if our findings were clinically significant, we correlated IL-5, IL-7, IL-9 and IFN-γ plasma levels with the patients' echocardiographic parameters, such as LVEF and LVIDd, and with disease duration.

Interestingly, in ICM patients, IL-9 levels inversely correlated with left ventricular ejection fraction (LVEF) (Table 2). Furthermore, IL-5 plasma levels inversely correlated with disease duration in NYHA III/IV ICM patients (Table 2). Neither IL-7 nor IFN γ instead correlated with echocardiographic parameters or disease duration, and no correlation was found between NIDCM patients and the above mentioned parameters. Further, no significant association was found between modulated cytokines and LVIDd (data not shown).

**Table 2:** Correlation between cytokine plasma levels and duration of disease or ejection fraction

Cytokine	IL-5	IL-9
Duration of disease	r = -0.475 (p = 0.05)	n.s.
Left Ventricular Ejection Fraction	n.s.	r = -0.364 (p = 0.02)

A negative Pearson's correlation coefficient (r) indicates that cytokine expression inversely correlates

with the variable in exam (e.g. : patients with longer disease duration had smaller concentrations of

plasmatic IL-5 in comparison with patients more recently diagnosed).

A statistically significant inverse correlation of IL-9 plasma levels with left ventricular ejection fraction

was found among the ICM group only (n = 42).

An inverse correlation between IL-5 plasma levels and duration of disease was found in NYHA III-IV

ICM patients only (n = 18).

## Discussion

The aim of this study was to further our understanding of the inflammatory pathways associated with CHF and to assess whether disease etiology affects cytokine cascades. To this end we analyzed several cytokines and growth factors' plasma levels with the XMAP technology. This tecnique has previously been used in cardiovascular research, specifically, in coronary artery disease, to evaluate selected cytokine ability in predicting long-term prognosis [23], and in Primary Graft Dysfunction [24].

In our study, we described two different modulation patterns, i.e. increased or decreased cytokine plasma levels, in chronic heart failure (ICM and NIDCM) patients as compared to controls. The first group was composed of MIP-1 β, MCP-1, IL-8, VEGF and IL-9, that were increased in both the ICM and NIDCM patients vs controls.

The second group was composed of IL-5, IL-7, IFN-γ, which showed lower plasma levels in both the ICM and NIDCM patients as compared to controls. Furthermore, IL-6 increased only in ICM while IL-1 β decreased only in NIDCM, both changes with respect to controls. Previous study results on IL-1 β systemic level changes in heart failure have been inconsistent and reported as either unchanged [25], increased [26], or decreased (present study) in such patients.

Several previous reports have described the role of cytokines and growth factors in the development and/or progression of chronic heart failure [1,5,9,11]; further, a number of such cytokines are regarded as disease biomarkers [17,18]. It is worth noting that our study, for the first time, has associated IL-5, IL-7, IL-9, and IFN-γ plasma levels with chronic heart failure.

It has been reported that IL-5 cardiac protein levels increase four days after Myocardial Infarction (MI) in a mouse model [27]; on the contrary, as yet no data is available on IL-5 serum or plasma levels in chronic heart failure patients. IL-7 is produced by stromal cells in lymphoid tissues and is required for T cell development and persistence in the periphery. Interestingly, local production of IL-7 has been associated with the maintenance and predominance of CD8+ T cells, which cause tissue damage in human chronic Chagas' disease cardiomyopathy, an inflammatory-dilated cardiomyopathy [28]. IL-9 is a mostly T-cell produced cytokine that has a functional role in allergic disease and resistance to intestinal nematodes [29], but no data is yet available on its role in heart disease. IFN-γ is an important pro-inflammatory cytokine produced by Th1 cells, that increases the expression of MHC class I and class II molecules. An increase in the percentage of IFN-γ-positive CD4 (+) T cells, has previously been described in patients with CHF [30]. Furthermore, an increase in IFN γ serum levels has also been described [31], in a mouse model, one week after MI, i.e. not in chronic heart failure experimental conditions.

Our study recorded decreased IL-5 plasma levels in both ICM and NIDCM groups vs controls. Interestingly, IL-5 plasma levels inversely correlated with disease duration in NYHA III/IV ICM patients. In fact, patients with longer disease duration had smaller concentrations of plasmatic IL-5 in comparison with patients more recently diagnosed. Furthermore, a statistically significant correlation with IL-5 plasma levels was found only among NYHA III/IV ICM patients, but not among patients with less severe chronic heart failure. This would suggest that decreased levels of this cytokine are associated with disease progression.

We here showed an increase in IL-9 levels in both ICM and NIDCM groups vs controls and that IL-9 levels inversely correlated with ejection fraction in ICM patients, but not in NIDCM patients. This would suggest that increased levels of this cytokine are associated with left ventricular dysfunction and, thus, with disease progression toward organ failure in ICM patients. Further, we reported a decrease in plasma IFN-γ levels and of IL-7 in both ICM and NIDCM CHF patients vs controls, but no correlation with echocardiographic parameters or duration of disease was found (not shown).

Nevertheless, our conclusions cannot broadly apply to the entire chronic heart failure population as only a small number of women were enrolled and analysed in both the ICM and NIDCM groups.

Interestingly, the cytokines that were identified in the present study are part of a gene network of expression/function regulation that link them to Norepinephrine, Endothelin-1, IL-1A, Galectin-3, TNFα, IL-6 and IL-18 [13], which are molecules associated with chronic heart failure [1,4,7,10,11,17,18]; thus we hypothesize that IL-5, IL-7, IL-9 and IFN-γ are part of signaling cascades that contribute to the development and/or progression of chronic heart failure.

Notwithstanding the ICM and NIDCM difference in pathogenetic causes there was a similar cytokine expression pattern when compared to controls. In fact, among all cytokines previously reported as being associated with CHF, our study showed only IL-6 and IL-1 β modification to be associated uniquely with ICM or NIDCM, respectively. All the other cytokines instead similarly increased or decreased, vs controls, regardless of the etiology. Further, we found that IL-1Ra was able to discriminate between the two patient groups, since it was statistically higher in the ICM group, although only a small increase was observed (data not shown).

In conclusion, the novelty in our study results is the increase of IL-9 and decrease of IL-5, IL-7, and IFN-γ plasma levels, in both ICM and NIDCM CHF patients vs controls and the inverse correlation of IL-5 and IL-9 plasma levels with duration of disease and LVEF, respectively, in ICM patients. Further studies are needed to assess whether the modification of IL-5, IL-7, IL-9 and IFN-γ plasma levels has a null, a protective, or a pathogenetic role in chronic heart failure and may constitute a potential target for therapeutic intervention.

## Competing interests

The authors declare that they have no competing interests.

This article conforms to ethics in the authorship and publishing of scientific articles [32].

## Authors' contributions

MN conceived the study design, coordinated the study and drafted the manuscript. CC isolated plasmas, performed statistical analysis and contributed to drafting the manuscript. LDV collected the clinical data of patients and performed statistical analysis. FC, GL, MCC and RM critically revised the manuscript's draft. GM performed statistical analysis, LS enrolled patients. EC and AF performed and interpreted the multiplex assay. All authors read and approved the final manuscript.
